# Estimating the Stochastic Bifurcation Structure of Cellular Networks

**DOI:** 10.1371/journal.pcbi.1000699

**Published:** 2010-03-05

**Authors:** Carl Song, Hilary Phenix, Vida Abedi, Matthew Scott, Brian P. Ingalls, Mads Kærn, Theodore J. Perkins

**Affiliations:** 1 Ottawa Hospital Research Institute, Ottawa, Ontario, Canada; 2 Department of Cellular and Molecular Medicine, University of Ottawa, Ottawa, Ontario, Canada; 3 Ottawa Institute of Systems Biology, University of Ottawa, Ottawa, Ontario, Canada; 4 Department of Applied Mathematics, University of Waterloo, Waterloo, Ontario, Canada; 5 Department of Physics, University of Ottawa, Ottawa, Ontario, Canada; 6 Department of Biochemistry, Microbiology and Immunology, University of Ottawa, Ottawa, Ontario, Canada; University of Virginia, United States of America

## Abstract

High throughput measurement of gene expression at single-cell resolution, combined with systematic perturbation of environmental or cellular variables, provides information that can be used to generate novel insight into the properties of gene regulatory networks by linking cellular responses to external parameters. In dynamical systems theory, this information is the subject of bifurcation analysis, which establishes how system-level behaviour changes as a function of parameter values within a given deterministic mathematical model. Since cellular networks are inherently noisy, we generalize the traditional bifurcation diagram of deterministic systems theory to stochastic dynamical systems. We demonstrate how statistical methods for density estimation, in particular, mixture density and conditional mixture density estimators, can be employed to establish empirical bifurcation diagrams describing the bistable genetic switch network controlling galactose utilization in yeast *Saccharomyces cerevisiae*. These approaches allow us to make novel qualitative and quantitative observations about the switching behavior of the galactose network, and provide a framework that might be useful to extract information needed for the development of quantitative network models.

## Author Summary

Decades ago, Waddington, and later Kauffman, likened the dynamics of a differentiating cell to a marble rolling downhill on bumpy terrain—the epigenetic landscape. In this metaphor, the valleys of the landscape represent the paths that cells can follow towards a stable cell type, and the fate of the cell is determined by the constant modulation of the epigenetic landscape by internal and external signals. With new technologies for measuring single-cell gene expression, it is increasingly feasible to map out these valleys and how external variables influence cellular responses. Moreover, it is possible to quantify population level effects, such as what fraction of a population of cells arrives at one valley or another, and variability at the cellular level, such as how individual cells bounce around within, and possibly between, valleys due to the stochasticity of cellular biochemistry. In this paper, we discuss which characteristics of the epigenetic landscape can readily be extracted from single-cell gene expression data, and describe computational methods for doing so.

## Introduction

One of the primary goals of systems biology is to uncover the dynamics of cellular networks. Sometimes, this has meant collecting time-series data and applying tools for time-series analysis such as Fourier methods to identify periodically expressed genes [1]–[3] or temporal clustering to identify different dynamic “modes” [4]–[6]. In other cases, it has meant the construction of explicit state-based dynamical models, based either on qualitative expectations of system behavior [7]–[10] or based more directly on quantitative experimental data [11],[12]. Another common goal has been to characterize the steady-state behavior of the network, which is of particular interest if the system exhibits multistability [13]–[15]. In these cases, the steady-states, along with their basins of attraction, have been likened to distinct cell types [16]–[18], and thus define the repertoire of “behaviors” available to the cell. Mathematically, the analysis of steady states falls into the domain of bifurcation theory, which addresses the existence, number and stability of fixed points or limit cycles/attractors of dynamical systems and how these change as a function of system parameters or inputs [19]. Usually, this analysis is performed on deterministic mathematical models such as differential equations or difference equations.

Here, we are concerned with the experimental and computational quantification of bifurcation-like behavior in stochastic genetic switches. There is considerable evidence that signalling networks in a population of genetically-identical cells exhibit large cell-to-cell variability in their output, despite operating in a homogeneous external environment (see e.g., [20],[21]). In some cases, inherent fluctuations in the internal state of the cells leads to distinguishable subpopulations, even when cells are genetically identical and experience a homogenous environment. For example, a ubiquitous network motif is the bistable genetic switch, with output variability distributed about high and low states dependent upon the level of an external input signal [13], [22]–[25]. Accurate estimates of bifurcation structure from noisy experimental can provide important qualitative, and in some cases quantitative, information about system behavior, guide model development and parameter estimation efforts, or help to discriminate among competing hypotheses regarding network architectures. For example, recent work has demonstated that the statistics of the fluctuations about the steady-states provides significant constraints on kinetic parameter estimation [26].

Two ingredients are necessary for empirical analysis of the bifurcation behavior of a cellular network. One is single-cell measurements of one or more cellular variables, such as gene expression. Technologies such as microarrays, SAGE or quantitative mass spectrometry, which operate on collections of cells or whole tissues, obscure potential heterogeneity in the sample. They do not discriminate, for example, between a 100% increase in expression of a gene, and a 200% increase in its expression in 50% of the cells. With technologies such as fluorescent cell imaging and flow cytometry, however, the state of each cell can be ascertained. As a result, one can determine whether the cell population is homogenous or if it comprises a set of subpopulations—each undergoing different dynamical behaviors corresponding to different growth strategies, differentiation endpoints, etc. The other necessary ingredient is a method for experimental manipulation of some system parameter(s) or environmental condition(s), in order to study how subpopulations change under varying conditions. This may mean changing the concentration of ligands or nutrients in the cellular environment or artificially manipulating the activity of regulatory factors inside individual cells. For example, Ozbudak et al. [13] recently used single-cell fluorescence microscopy to establish an empirical map of the two-dimensional bifurcation diagram for the lactose utilization network in *Eschericia coli* as a function of the systematic variation of two environmental parameters. Moreover, targeted disruption of feedback loops within the galactose utilization network of *Saccharomyces cerevisiae* has provided key insights into the control of cell-cell variability in gene expression and mechanisms underlying the stochastic switching between distinct epigenetic expression states [25],[27]. Increased use of these techniques demands the establishment of methods for analyzing the generated data in a statistically robust and computationally efficient manner.

The organization of this paper is as follows. First, we discuss traditional bifurcation analysis in greater detail, introducing in particular saddle-node bifurcations, a type of bifurcation widely associated with the dynamics of gene regulatory switches. We then describe the necessity of generalizing the notion of bifurcation behavior to account for the inherent noise (stochasticity) in cellular networks. Next, we present the data that motivated our study—single-cell flow cytometry data measuring activity in the yeast galactose utilization network over a range of extracellular galactose concentrations. We then report on two broad approaches to analyzing this data and extracting estimates of bifurcation structure, namely, mixture density modeling and conditional mixture density modeling. We evaluate the relative strengths of these approaches, and describe a number of novel qualitative and quantitative observations about switching in the galactose network.

## Results

### Stochastic bifurcation structure

Bifurcation analysis is a branch of dynamical systems theory concerned with steady-state or asymptotic behaviors of a dynamical system [19]. Typically, bifurcation analysis is applied to a deterministic dynamical model, such as a system of difference equations or differential equations. To give a concrete example inspired by the data presented and analyzed later in this paper, imagine a situation where a single gene is activated by an input signal 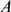, representing, for example, the activity of transcription factor protein. Let 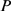 denote the gene's protein product. Suppose that the gene is an auto-activator: the protein product acts as a transcription factor to upregulate its own expression. Following standard modeling approaches (e.g. [28]) we describe the time-varying behaviour of the protein abundance by the differential equation
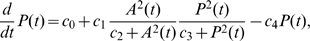
(1)where the parameter 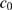 corresponds to a basal level of protein production, 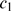 is the maximal additional production attributable to regulation, 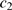 and 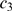 characterize the effects of the activators, and 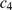 indicates the rate of protein degradation or dilution due to cell growth.

**Figure 1:**
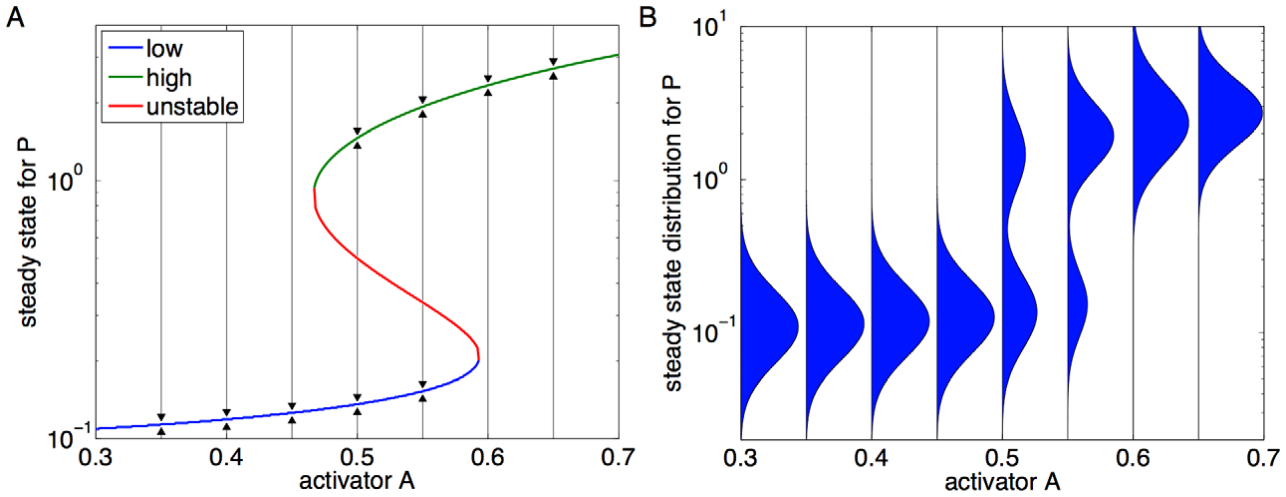
Examples of bifurcation behavior. (A) Bifurcation diagram of the system in Equation 1, an idealized model of a gene activated by signal 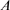 as well as by its own protein product 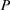, with parameters 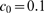, 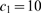, 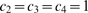. The three colored curves identify low, high, and unstable steady states for 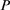 (i.e., values for which 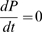), as a function of the activating input 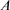. Black arrows show the direction of change of 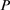, assuming 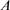 constant. (B) With noise in the dynamics, individual cells would fluctuate in the vicinity of the steady states, leading to some overall distribution for 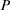 over time or across cells.

Figure 1A is a bifurcation diagram for this system, showing the steady state values of 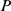 as a function of the input 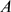, which in this context is called the bifurcation parameter. Intuitively, if levels of 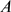 are low, then little 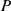 is produced and the system reaches a steady state at a low level of 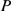. Conversely, if 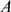 is highly abundant, then a great deal of 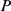 is produced, leading to a high steady state. Most interestingly, when 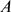 lies in and intermediate range, three steady states coexist. Intermediate levels of 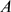 and a large initial amount of 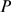 will stimulate sufficient production to maintain 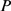 at a high concentration. However, if initially the level of 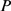 is low, production is not maintained, and the system reaches a low steady state. There is also a third, unstable steady state between the low and high steady states. The values of 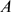 at which the number of steady states changes, i.e., the turns of the ‘S’-shaped curve in Figure 1, are called *bifurcation points* and correspond in a deterministic system to the critical values of 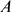 where a small change in this parameter may cause the system to transition between states of low and high levels of 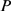.

In contrast with deterministic models, real cellular networks can be significantly noisy, with system variables fluctuating over time for a variety of reasons, including, for example, fluctuations in biochemical reaction rates, random partitioning of cellular content at cell division, and variation in cell size and cell age (see e.g., [21]). Thus, if one were to observe multiple instances of a bistable system—say, a culture of genetically identical cells experiencing a homogeneous medium—one would not expect the experimental measurements to agree with the predictions of a deterministic model, even after the culture has attained a steady behaviour [29]. Noticeably, stochastic fluctuations will constantly push individual cells on excursions away from a stable expression state, causing a broadening of the population distribution around this state. The mean of the population distribution will reflect the steady state expression only when these excursions are symmetric, and the mode of the distribution, which corresponds to the state where the system on average spends most time, may be the better surrogate of deterministic steady states in a stochastic dynamical system (e.g., [30]).

**Figure 2:**
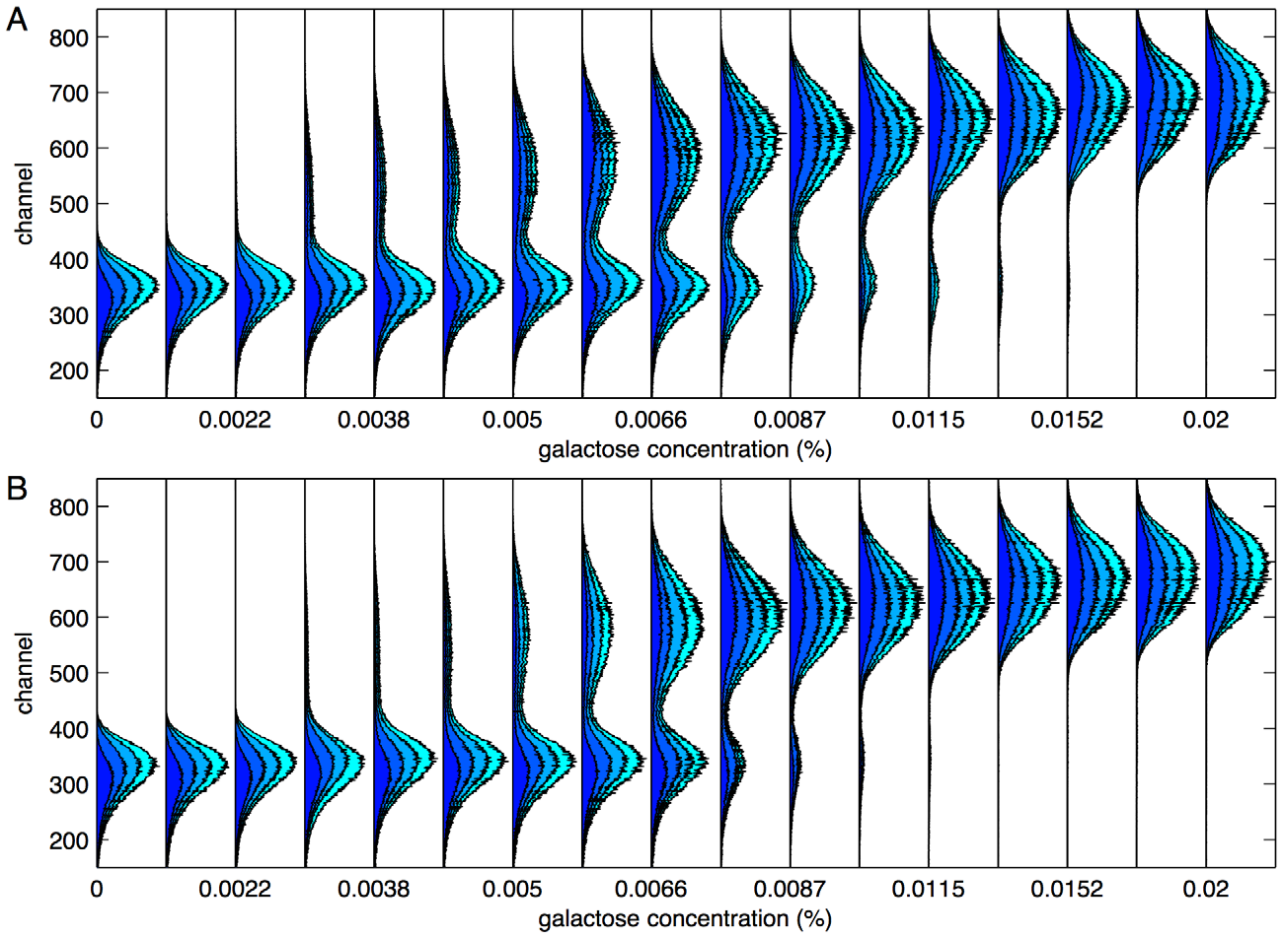
Fluorescence data for the reporter protein indicating activity level of the galactose utilization network in *S. cerevisiae*. Fluorescence is reported as a function of galactose level in culture (expressed as percent weight per volume; 1% = 10g/L), under the galactose pregrowth condition (A), and the raffinose pregrowth condition (B). All four biological replicates are shown stacked on each other. The blue area represents the number of cells counted in each fluorescence channel in replicate 1, the next lighter blue area is the sum of the counts in the first two replicates, and so on.

In a bistable system, fluctuations can induce stochastic transitions between the two expression states such that some cells are expressing at low level while others express at high levels. The result is the emergence of a bimodal population distribution and subpopulations with distinct expression characteristics. Figure 1B depicts what the steady state distribution for 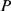 might look like as a function of 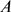, assuming the stochastic system would show a lognormal distribution for 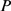 about the deterministic steady states. (For a graph of real data from that galactose network, see Figure 2.) In this case, the time-invariant steady state distribution of the system is reached when the probability that a cell will switch from the low to the high expression state is the same as that associated with a transition from the high to the low expression state. The time it takes for the system relax to steady state, which is set by the kinetic rate parameters and the level of noise in the system, can range from the order of seconds to several tens of cell generations [31]. It is also noted that very rapid transitions between expression states may result, at the population level, in a persistent subpopulation that is not associated with a steady state in the deterministic model, and that noise, under certain conditions, may shift the location of bifurcation points or induce new bifurcations (see e.g. [32]).

How can we capture the bifurcation behavior of a stochastic dynamical system? Suppose that 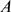 represents the bifurcation parameter (e.g., an externally controlled parameter or variable), and 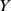 represents an observed variable of the system, such as the protein abundance. Suppose that for any value of 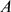, and under a specified set of experimental conditions, we observe a population of cells with values of 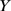 following some distribution 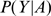. We propose that the stochastic bifurcation structure of the system should specify four pieces of information as a function of the parameter 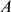:

1. The number of distinguishable subpopulations
2. Some notion of the “location” of those subpopulations, in terms of the observable variable 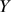

3. Some notion of the variability in 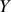 within each subpopulation
4. The fractions of the whole population that are represented by each subpopulation

This is not a formal definition of stochastic bifurcation structure; these are principles, which might be formalized in a number of different ways. For example, as mentioned above, the modes of the steady state distribution of a stochastic dynamical system have previously been proposed as analogs to the steady states of a deterministic model. Thus, one might use the modes of the distribution 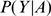 to determine the number and location of subpopulations, satisfying the first two parts of the definition above. In particular, one could use bimodality as a *defining feature* of bistability in a stochastic switching system and associate bifurcation points with parameter values 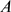 where the population distributions change from unimodal to bimodal. In many cases, this may work well, although below we will show some reason to question the use of modes as defining of the number of subpopulations.

If one can assign every cell to a subpopulation, then the variance of 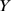 within each subpopulation and the relative sizes of the subpopulations provide natural answers to the third and fourth parts of the definition above. As with the locations of the modes, these features of the stochastic bifurcation structure may be related to properties of a deterministic model. For example, the degree of variation around a mode, or the fraction of time the system spends near the mode, are related to the degree of stability of the state in the deterministic model [32]. Below, we use the formalism of mixture models to instantiate these four principles of stochastic bifurcation structure. First, however, we present our experimental data on the galactose network.

### The galactose utilization network in *S. cerevisiae*

Our thoughts on stochastic bifurcation structure and methods to estimate it were motivated, indeed necessitated, by data we collected on activity in the galactose utilization network in *S. cerevisiae*. The network includes genes for the import and metabolism of galactose as well as various regulatory genes [33],[34], and is known to behave as a bistable switching network. For a range of external galactose concentrations, cells stochastically switch between induced and non-induced states [25]. To assay this behavior, a standard laboratory strain was augmented with a gene encoding a fluorescent protein under the control of the promoter region normally regulating the transcription of the endogenous *Gal10* gene (see Materials and Methods). *Gal10* is a general indicator of activation of the network, hence the fluorescent reporter should be expressed when and only when the native network is itself active. Cells were cultured for 22 hours in 17 different constant concentrations of galactose from two different initial conditions—pregrowth in the absence of galactose to establish a non-induced initial state or pregrowth in the presence of galactose at high concentration to establish an initial state where all cells are induced. The activity of the network in individual cells was quantified by flow cytometry to measure the intensity of the fluorescence emitted by the expressed reporter gene. Four biological replicates were made of every experiment. The collected data comprises counts of how many cells were detected in each of 1024 fluorescence channels, which are logarithmically related to real fluorescence intensity and have a dynamical range of four orders of magnitude (i.e., channel 1024 represents 10,000 times the intensity of channel 1).

Figure 2 displays the data, which is broadly consistent with previous experiments [25]. At low galactose levels, all cells show low network activity. At higher galactose concentrations, a highly active subpopulation emerges, and at yet higher levels, the highly active subpopulation dominates and the low-activity subpopulation disappears. While these overall trends in the data are visually clear, the challenges in analyzing the data quantitatively include robustly determining the locations and sizes of the subpopulations, especially when one is much smaller than the other, dealing with cells not clearly attributable to any one subpopulation, and separating cell-to-cell variability from replicate-to-replicate variability. Ideally, these should be done in a statistically robust, computationally simple, and objective manner.

### Estimates of stochastic bifurcation structure of the galactose network

#### Mixture models and conditional mixture models

A natural approach to modeling multi-modal data is to employ mixture distributions. We model data from each biological replicate separately, in order to avoid conflating replicate-to-replicate variation with cell-to-cell variation within a replicate. Consider a replicate, 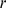, and a galactose concentration, 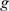. A mixture distribution expresses the probability that a particular cell is detected in fluorescence channel 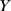 in terms of 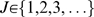 component distributions as
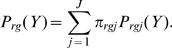
(2)Here, 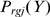, the 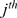 component in the mixture, is typically some elementary probability density, such as a normal, lognormal, exponential, or uniform distribution. The values 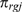, variously called “mixture coefficients”, “component weights” or “prior probabilities”, specify the degree to which the 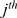 component contributes to the overall distribution. For every 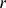 and 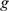, they must be positive and must sum to one.

For a given replicate, fitting mixture distributions to each galactose concentration 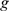 meets most of our requirements for specifying stochastic bifurcation structure. If we assume each component of the distribution corresponds to a meaningful subpopulation in the data, then the number of components (which must be optimized as part of the model fitting) tells us the number of subpopulations. The mixture coefficients tell us the relative sizes of those populations. Looking at a component distribution, 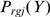, the mean, median or mode can be used to define the “location” of the subpopulation. We will use Gaussian components to represent subpopulations, in which case the mean, median and mode are the same. Finally, the variance of the component distribution represents variability within the subpopulation.

The only downside of this approach is that it does not explicitly model the dependence of these features of stochastic bifurcation structure on the external controllable bifurcation parameter—the galactose concentration 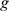. Rather, it gives us “snapshots” of the stochastic bifurcation structure at the particular galactose concentrations for which data is collected. As a result, it does not immediately offer a means to predict the fluorescence distribution one would see at a different, untested galactose concentration—though certainly some such predictor could be constructed post hoc from the set of mixture distributions estimated at each measured concentration. Because most aspects of stochastic bifurcation structure might be expected to vary smoothly with 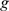, it makes sense to make the dependence on 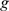 explicit. For this reason, we explored conditional mixture models.

For a given replicate 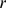, a conditional mixture model expresses the probability that a cell is detected in fluorescence channel 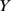 conditioned on any possible galactose concentration 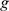 as:
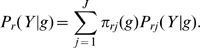
(3)The difference between this and the previous equation is that mixture coefficients, 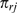, are now functions of 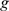, as are the component distributions, 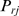. For example, if the component distributions are Gaussian, we may represent the dependence on 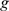 by assuming some smooth functional form for the means and variances of those Gaussians as a function of 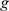. If a conditional mixture model is fit based on measurements at certain galactose levels, it can be evaluated to predict a distribution for 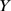 at different concentrations. Such models also tend to represent data much more compactly—that is, with fewer parameters—than a set of (unconditional) mixture models, which keep separate parameters for each level of 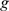 modeled.

#### Modeling assumptions and fitting approaches

We used two different approaches to fit mixture models and one approach to fit a conditional mixture model to the data. In all approaches, the mixtures contained one or more Gaussian components as well as a single uniform component. The uniform component was given a fixed mixture coefficient of 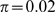, and was used to account for inevitable outliers in the data arising, for example, from contaminating particles or carry-over between samples. In our first approach to fitting mixture models, we used the standard expectation-maximization (EM) algorithm [35] to fit the model parameters (i.e., the mixture coefficients and the Gaussian means and variances) at each galactose concentration. Model parameters were taken as the best-fitting (highest log likelihood of the data) out of 100 runs of EM from different random initial conditions. We first fit a model with one Gaussian component, then two, then three, etc., until cross-validation estimated that additional Gaussian components were not significantly improving the fit. Details are in the Materials and Methods section. In our second approach to fitting mixture models, we used a mode estimation technique to identify peaks in the data. For each mode identified, we introduced one Gaussian component to the mixture, with mean equal to the mode location. We then used EM to fit the mixture coefficients and variances of the Gaussians, leaving the means fixed. For the conditional mixture models of each replicate, we assume two Gaussian components in addition to the uniform component, to account for the low-expressing and high-expressing subpopulations. The means of the Gaussian components were assumed to be affine in the galactose concentration 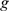.
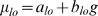
(4)

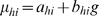
(5)The variances of the Gaussian components were assumed independent of 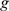. For the mixture coefficients, we assume the weight of the low-expressing component took the form
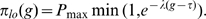
(6)This function is equal to 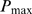 for galactose concentrations below the threshold 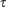, above which the function decays exponentially towards zero at rate 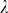. The rationale for this particular form was based on observations from our unconditional mixture fits, and will become clear shortly.

#### Modeling results

**Figure 3:**
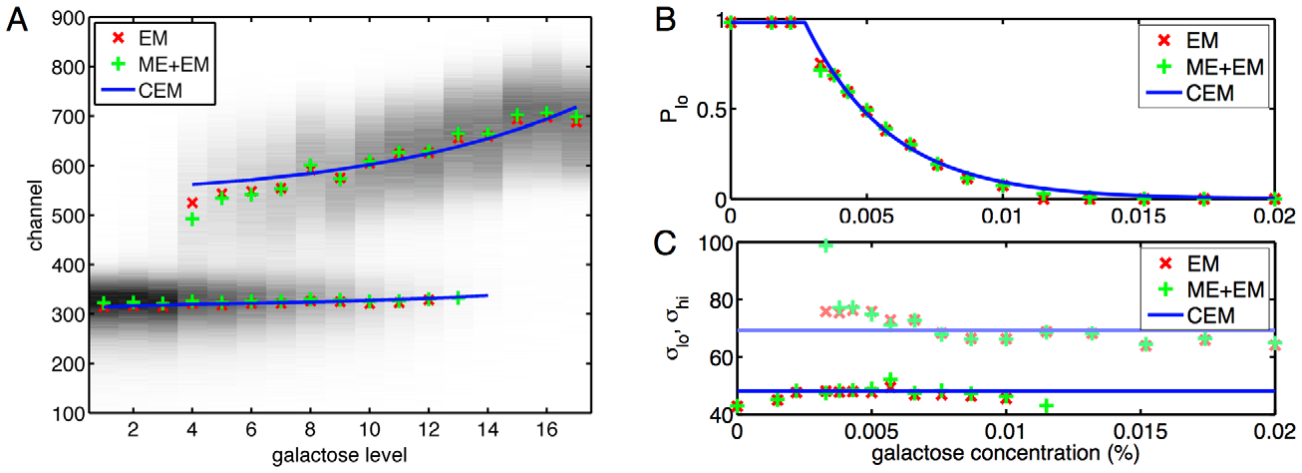
Results of mixture modeling on replicate one. (A) Means of subpopulations, as extracted by: mixture models estimated by the expectation-maximization algorithm (EM), mixture models estimated by a combination of mode estimation and expectation-maximization (ME+EM), and a conditional mixture model estimated by expectation-maximization (CEM). The x-axis represents the 17 levels of galactose tested, in order of increasing concentration. The y-axis represents fluorescence channels of the flow cytometer, which are proportional to the logarithm of fluorescent intensity. Darker background shading represents more cells counted in the channel at the given galactose level. (B) Estimated mixture coefficients (prior probabilities) of the low subpopulation as a function of galactose concentration. (C) Estimated standard deviations of the Gaussian distributions representing low (darker) and high (lighter) subpopulations as a function of galactose concentration.

Figure 3A shows the locations of the subpopulations in replicate one, as estimated by the three methods: mixture models fit by EM (EM), mixture models fit by mode estimation followed by EM (ME+EM), and conditional mixture models fit by EM (CEM). There is strong agreement between the methods in terms of both the number and location of the subpopulations. Activity of the low subpopulation, when it exists, appears nearly independent of 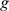. However, the location of the high subpopulation increases with increasing galactose concentration. All methods agree that a distinct high subpopulation is established at the fourth galactose concentration (0.0033%), though the methods disagreed on this feature in other replicates, as we will show shortly. There is minor disagreement on the galactose concentration at which the low subpopulation disappears. For the conditional mixture model, we have plotted the low subpopulation mean as long as its mixture coefficient is greater than 0.01. (Due to the form of the model used for mixture coefficients, the model actually assumes the low component exists at all galactose concentrations, though with size that vanishes exponentially as a function of increasing concentration.)

Figure 3B shows the estimated mixture coefficients for the low subpopulation as a function of galactose concentration. The mixture coefficient for the high subpopulation, where it exists, is 0.98 minus the low mixture coefficient, as the coefficients for the low, high and uniform components must sum to one. At the lowest galactose levels, virtually all of the cells are in a low-expressing state. Then, apparently abruptly, a high subpopulation becomes established and comes to dominate with increasing galactose concentration, as the low subpopulation fades away. It was the close agreement of the unconditional mixture models on this basic story that motivated the form of Equation 6 for the mixture probability of the low subpopulation in the conditional mixture model.

Figure 3C shows the standard deviations estimated by the three methods. All methods find that variability within the high subpopulation is greater than within the low subpopulation—a feature readily visible in the data (e.g., Figure 3A). Otherwise, there appears to be little dependence on galactose concentration, except perhaps for a slight decrease in variability within the high subpopulation with increasing galactose.

**Figure 4:**
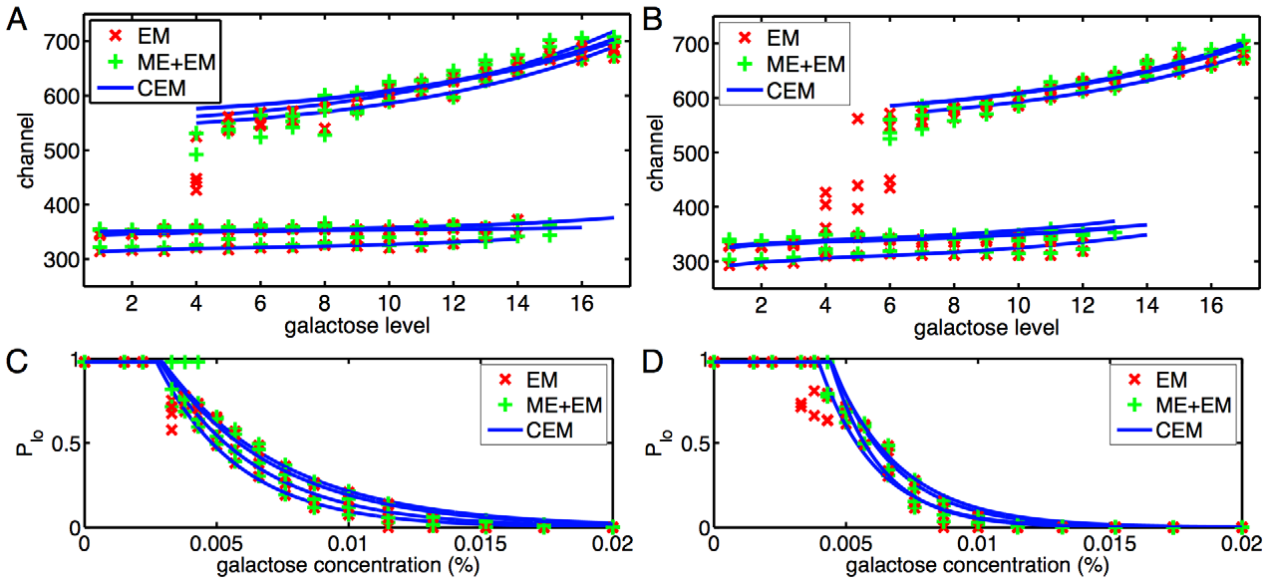
Comparison of subpopulation means and sizes across replicates. (A) Subpopulation means as extracted by the three fitting methods, in all four replicates of the gal-pregrowth condition. (B) Subpopulation means in the four raf-pregrowth replicates. (C,D) Estimated sizes of the low subpopulations in the gal-pregrowth and raf-pregrowth conditions respectively.

**Figure 5:**
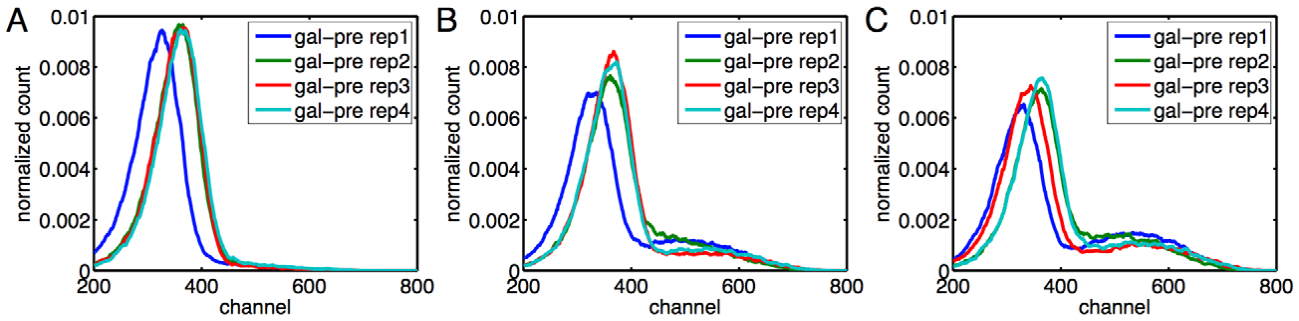
Emergence of the high subpopulation at increasing galactose concentrations, in the galactose pre-growth condition. Empirical count distributions for the four replicates are shown, smoothed using a width-11 moving average to improve visibility. (A) At the third galactose concentration (0.0022%). (B) At the fourth galactose concentration (0.0033%). (C) At the fifth galactose concentration (0.0038%).

**Figure 6:**
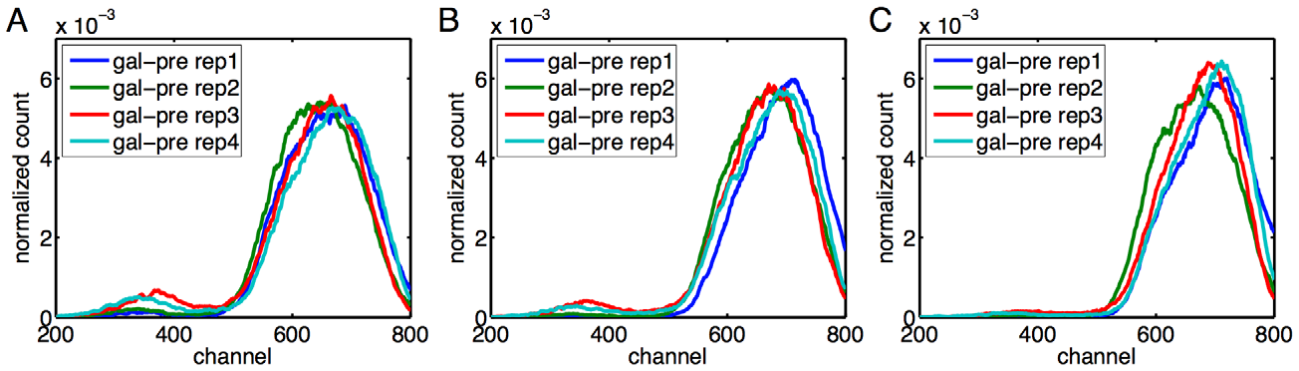
Disappearance of the low subpopulation at higher galactose concentrations, in the galactose pre-growth condition. Empirical count distributions for the four replicates are shown, smoothed using a width-11 moving average to improve visibility. (A) At the 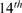 galactose concentration (0.0132%). (B) At the 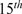 galactose concentration (0.0152%). (C) At the 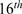 galactose concentration (0.0174%).

In Figure 4A and B we show the subpopulation means estimated by the three methods in the galactose pregrowth condition and the raffinose pregrowth condition respectively. In panels C and D, we show the estimated subpopulation sizes. Many qualitative features observed in replicate one with galactose pregrowth continue to hold. The expression in the low subpopulation is largely independent of galactose concentration, whereas expression in the high subpopulation increases with galactose concentration. There is exclusively a low subpopulation up to some galactose concentration, above which a high subpopulation is abruptly established and grows gradually with increasing galactose as the low subpopulation fades away. However, there are several differences between the replicates. A key difference is in the establishment of the high subpopulation. In the galactose pregrowth condition, there is disagreement as to whether a high subpopulation exists at the fourth galactose level. All the EM and CEM fits concluded that there is a high subpopulation, but only two of the ME+EM fits did so. Moreover, there is disagreement as to the location of those subpopulations, with the EM fits reporting lower-expressing high subpopulations than the other methods. A close look at the data (Figure 5) helps to illuminate these discrepancies. Panels A and C show that at the third and fifth galactose concentrations tested, all replicates have, respectively, no high subpopulations and clear high subpopulations. At the intermediate concentration, all replicates show an emerging high subpopulation. In some of the replicates, this subpopulation is sufficiently blended with the main low subpopulation so that there is no distinct peak. This is why the ME+EM approach, which determines the number and location of subcomponents by peak detection, does not identify a high subpopulation in some replicates. The fact that the high subpopulation tends to have much higher variance than the low subpopulation increases the difficulty of distinguishing the two, as well as pinning down the location of the high subpopulation. A similar phenomenon occurs in the raffinose pregrowth condition (Figure 4B,D), though over a slightly higher and broader range of galactose concentrations. The disappearance of the low subpopulation at yet higher galactose levels does not show the same indistinct blending of high and low subpopulations (Figure 6). Rather, the low subpopulation remains separate from the high subpopulation while shrinking in size.

**Figure 7:**
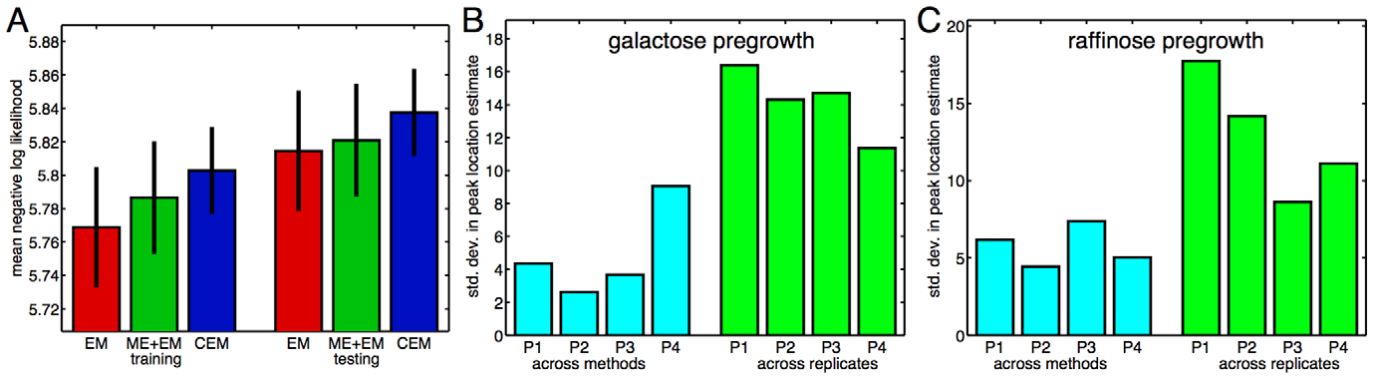
Comparison of goodness-of-fit between methods and biological replicates. (A) For each method, the mean negative log likelihood of the data. “Training” means each model is evaluated on the same data to which it is fit, whereas “testing” means each model is evaluated on the data from the other three replicates having the same pregrowth condition. Black bars indicate 95% confidence intervals. (B,C) Variability in the estimated locations of four subpopulations: the low (and only) subpopulation at the zero galactose concentration (P1), the low subpopulation at the 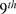 galactose concentration (P2), the high subpopulation at the 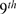 galactose concentration (P3), the high (and only) subpopulation at the largest tested galactose concentration (P4). Cyan bars show the variability attributed to different estimation methods, whereas green bars show the variability attributed to different biological repliciates.

While the three fitting methods produce qualitatively similar results in many respects, a question arises as to whether any of the methods is better than the others in a quantitative sense. The first way we examined this question was to compare the log likelihood of the data under different models and replicates. Figure 7A shows the mean negative log likelihood (see Materials and Methods for exact definition) that each model achieved on the fitted data (“training error”), and when evaluated on the data from other replicates (“testing error”). As is often the case, the training errors are smaller than the testing errors. The results show a potential trend for the EM fits to be better than the ME+EM fits, and for the ME+EM fits to be better than the CEM fits. However, none of the pairwise differences in testing error reach statistical significance at the 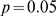 level.

In Figure 7B,C we attempt to separate the degree of disagreement between methods and inherent variability between replicates. We examined estimated locations of four different subpopulations, as specified in the figure caption. For each subpopulation, we estimated biological variability by averaging the three location estimates (one from each method) and computing the standard deviation of that pooled estimate across the four replicates in the same pregrowth condition. To estimate variability due to each method we did the reverse—averaging each method's location estimates across replicates, and then taking the standard deviation across the three methods. In both pregrowth conditions and for all four subpopulations, the variability across replicates was significantly greater than the variability among the estimates of the different methods. One-way ANOVAs of the location estimates for each subpopulation result in a similar conclusion (data not shown).

## Discussion

We have defined a notion of stochastic bifurcation structure suitable for studying the behavior of stochastic genetic switches, and we have generated an extensive map of the response of the canonical bistable yeast galactose utilization network to variation in external galactose concentrations. While the data broadly conforms to our expectations for stochastic switching between low and high expression states within the network, several additional properties are noteworthy. The establishment of a “high” expressing subpopulation occurs rather abruptly and fairly consistently at a concentration of approximately 0.003% galactose, although this state is initially overlapping the low expressing subpopulation. By contrast, the low subpopulation fades away more gradually at higher concentrations, while maintaining clear separation from the high subpopulation. Activity within the high subpopulation, in terms of fluorescent intensity, increases substantially as a function of galactose concentration—by approximately 300% over the range of concentrations tested. Activity within the low subpopulation is fairly constant, and is, in most cases, indistinguishable from that of cells not expressing the reporter gene (data not shown), though there may be a mild increase in expression as the galactose concentration increases. Hence, the response of the network to varying conditions appears to combine a boolean-type “binary” switch between “on” and “off” expression states with a continuous “graded” modulation of activity within the “on” state.

From a methodological point of view, we proposed that mixture density estimation and conditional mixture density estimation are ideally suited to extracting stochastic bifurcation structure from real, noisy data. Our tests of two different mixture fitting methods and one conditional mixture fitting method suggested that, in most respects, the methods are equally accurate in fitting the data. It is possible that the conditional mixture model was less accurate. Visually, it appears to overestimate the location of the high subpopulation at smaller galactose concentrations, and underestimate it at higher concentrations (see Figures 3A or 4A,B). However, this is due simply to the affine form assumed for the dependence of subpopulation location on concentration. Alternative forms could readily be chosen to allow greater flexibility in fitting the data. Regardless, the overall level of disagreement between methods appeared smaller than the variability between different biological replicates. One potentially important distinction between the two mixture modeling approaches, standard EM and mode estimation followed by EM, is that the former is able to identify a “high” subpopulation at lower galactose concentrations than the second approach. This is because, at the lowest galactose concentrations, the “high” subpopulation is very broad and partially merged with the typical low subpopulation—in some cases, to such a degree that the overall distribution is still unimodal (see Figure 5). The standard EM method, because it requires multiple runs to avoid the problems of local minima and for cross-validation, is considerable slower than either of the other methods. Still, all methods run orders of magnitude faster than the data collection takes, so this is a minor concern.

Conditional mixture models have several additional advantages compared to fitting the data at each galactose level separately: they use fewer total parameters, and are thus less likely to overfit the data, and they explicitly represent and make predictions for the bifurcation structure at all values of the bifurcation parameter—not only the values tested experimentally. This approach worked well on our data. The drawback of this approach is that it requires choosing functional forms to represent the dependence of mixture probabilities and mixture component parameters on the bifurcation parameter. In this case, a proper means of representing mixture probabilities only became clear after doing the individual fits. In early conditional mixture model fits, we assumed the mixture probabilities were independent of galactose concentration. This had the unfortunate side affect that the high component would start to “capture” cells from the low subpopulation at low galactose levels, dragging down the whole mean curve for the high subpopulation until it intersected and overlapped with the low subpopulation. The form we chose for the mixture probabilities avoids this problem by definitively assigning cells to the low component at all galactose levels below some threshold. This illustrates that the strength of using few parameters and explicitly generalizing across bifurcation parameter values also implies a danger of poor performance if an inappropriate representation is chosen. While this is a truism in the statistics and machine learning communities, it is all the more important to keep in mind in systems biology where there is a greater focus on interpreting models, as opposed to, say, being concerned only about prediction accuracy.

Despite our focus on mixture modeling, one can imagine other approaches for estimating stochastic bifurcation structure. For example, clustering methods such as K-means or self-organizing maps could readily be applied in much the same way as we applied mixture density estimation. Nonparametric density estimation techniques might also be applied, although it would take extra effort to extract subpopulations from a nonparametric density estimate. Investigating such alternative approaches is an important topic for future research.

Part of our contribution is in specifying four types of information that should be included in a stochastic bifurcation analysis: the number of distinct subpopulations, the fraction of cells they contain, the level of expression and the variance within each subpopulation. Our notion of stochastic bifurcation structure is considerably different from ideas employed in stochastic bifurcation theory, which addresses the behavior of explicitly stochastic dynamical models, such as stochastic differential equations [36]. The primary concern of stochastic bifurcation theory is the number and stability of different steady state distributions of a model. In gene regulatory networks, it is not unreasonable to assume that any given cell could eventually, through random fluctuations, reach the same state as any other cell [23],[25]. Such a system is said to be “communicating”, and under fairly general conditions, has only a single steady state distribution for each bifurcation parameter value [37]. By the gross standard of stochastic bifurcation theory, such a system does not show any bifurcations at all. We, by contrast, have attempted to paint a finer-grained picture of the dependence of a stochastic dynamical system on an experimentally manipulated parameter. This picture is largely consistent with the expectation that fluctuations within the context of a deterministic network model constantly push individual cells on excursions away from a stable expression state, and induce stochastic transitions between the two expression states to generate bimodal population distributions [29]. Indeed, our focus identifying subpopulations is closely related to the idea in Kepler and Elston [29] of defining bifurcations via the number of critical points in the steady state distribution. However, our approach is much different; whereas they start with first-principles stochastic chemical descriptions of simple gene regulatory models, we start with empirical measurements of a complex gene regulatory system.

Stochastic bifurcation structure may provide useful information for the development of quantitative regulatory network models, however this remains to be investigated. The exact relationship between stochastic observables and model features is not yet clearly established. For example, models of gene regulatory networks are usually derived from molecular interactions within individual cells and rarely consider effects due to population dynamics. The gradual fading of the low-expressing subpopulation observed in our experiments could be due the stochastic dynamics of the regulatory network itself, or it could be due to a reduced growth rate of the low-expressing cells. Additionally, while we took steps to present the cells in each culture with homogenous extracellular conditions (see Materials and Methods), it is likely that there was some variability in the conditions experienced by different cells or by the same cell over time. Depending on the magnitude of this effect, it too might need to be estimated, if possible, and separated from intrinsic cell-to-cell variability if one wants accurate estimates of cellular network parameters.

Careful quantitative estimation of stochastic bifurcation structure facilitates comparison between different experimental conditions or genetic backgrounds. For example, the yeast strain studied by Acar *et al.* (W303) is much less sensitive to galactose and displays an almost 10-fold shift of the bimodal region (to concentrations between approximately 0.02% and 0.3%) compared to our strain (an equivalent of BY4743; see also discussion in Bennett *et al.* [38]). Thus, even subtle differences in DNA-encoded parameters may have significant impact on the stochastic bifurcation structure of a given gene regulatory network. It should be possible to link DNA sequence information to quantitative properties of gene regulatory networks. This may require the development of several methodological, in addition to experimental, approaches that can extract consistent information about stochastic bifurcation structures. For example, it would be necessary to compare different, empirically-measured stochastic bifurcation structures associated with different genotypes to determine whether there is a statistically significant difference between them and, if so, identify the origin of the difference using a dynamical systems theory or other type of modelling framework. In addition, such methods could be useful to investigate how gene regulatory networks have evolved, to infer regulatory relationships between genes, or refine our knowledge of them, based on stochastic bifurcation behavior in experiments involving systematic genetic perturbations, such as gene deletions, gene knockdown or overexpression experiments.

## Materials and Methods

### Strains, growth conditions, and gene expression assays

The experiments use a diploid *Saccharomyces cerevisiae* strain expressing a single copy of yeast-enhanced green fluorescent protein (*yEFPG*) from the native promoter of the GAL10 gene (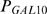). The diploid was obtained by mating two haploid strains, a Mat a strain (yHP101) derived from BY4741 (Mat a, 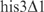; 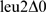; 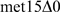; 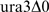, Open Biosystems) by PCR-mediated replacement of the open reading frame of the *Ade2* gene by a *Leu2* expression cassette, and a Mat 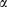 strain (yHP201) derived from BY4742 (Mat 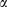; 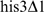; 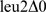; 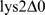; 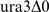, Open Biosystems) by PCR-mediated gene replacement of *Ade2* by a DNA fragment carrying the reporter cassette 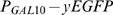 and an expression cassette conferring histidine auxotrophy. Following PCR validation of the appropriate gene replacements, the diploid strain, designated yHP301 (Mat 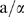, 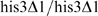; 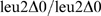; 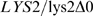; 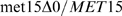; 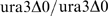; 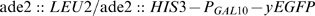) was stored at 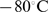 in rich media (YPD) containing 20 g/L Yeast Bacto-Peptone (Wisent), 10 g/L yeast extract (Wisent) 20 g/L glucose (Sigma-Aldrich) and 1% w/vol adenine (Sigma-Aldrich) supplemented with 15% w/vol glycerol (Sigma-Aldrich).

Prior to quantification, yHP301 was streaked onto synthetic dropout medium (Wisent, Inc.) agar plates without leucine and histidine supplemented with 2% w/vol glucose and 1% w/vol adenine. Individual colonies were used to inoculate 3 mL rich media (YPR) containing 20 g/L Yeast Bacto-Peptone, 10 g/L yeast extract, 1% w/vol adenine and 2% w/vol raffinose (Wisent) or YPR media supplemented with 2% w/vol galactose (Becton, Dickenson). Following growth for 24 hours at 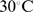 and continuous shaking (250rpm), twenty-one 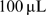 aliquots of each culture were transferred to a deep well block and washed twice with 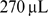 YPR media supplemented with varying amounts of galactose (final concentrations 0.0, 0.0015, 0.0022, 0.0033, 0.0038, 0.0043, 0.0050, 0.0057, 0.0066, 0.0076, 0.0087, 0.0100, 0.0115, 0.0132, 0.0174, 0.020, 0.080, 0.20, 0.50, 2.0%w/vol). Following the wash, cells were resuspended in 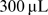 of the appropriate media and optical density (OD) quantified with a Perkin Elmer Victor3V plate reader using 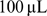 cultures. A fraction of the remaining volume was subsequently used to inoculate 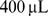 fresh media containing the appropriate amount of galactose to an OD of 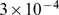, and grown in a 96 deep well block for 22 hours at 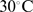 and 250rpm prior to analysis.

Reporter gene expression was quantified in individual cells using a Beckman-Coulter FC500 flow cytometer. A total of 60,000 events were collected for each condition and filtered using custom-written software script using a fixed elliptical forward/side-scatter autogate capturing approximately 50% of the events in each sample. The fluorescence intensity (488nm excitation, 510–550nm emission) associated with these events was used to generate representative expression distributions for each sample condition. A total of four replicates were obtained, for each final galactose concentration and both pre-growth conditions.

### Mixture density estimation

Mixture density estimation using EM used 100 runs in an effort to avoid problems with stopping at solutions that were only locally optimal. Each of the 100 runs began from different random initial parameters. The means of each Gaussian component were chosen uniformly between the lowest and highest data point. Standard deviations were initialized to 50—roughly the level observed at single-subpopulation galactose concentrations—and initial mixture probabilities for the Gaussians were set to 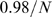 where 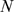 is the number of Gaussians. (Recall that a fixed 0.02-weighted uniform density is also part of the mixture). The exception to this rule was the mode-estimation-plus-EM approach, for which means were initialized to the mode estimates, and we used a single run of EM. The parameter updates during the M-step were as described, e.g., in Bishop [35]. If the variance of a Gaussian shrank below 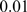, the component was eliminated, because such a Gaussian is focussed on a single fluorescence channel, and does not represent a true subpopulation.

The EM fitting employed cross-validation to determine the proper number of Gaussian components to have in the mixture for each replicate and at each galactose level. After fitting a model with 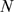 Gaussian components, we tested whether an 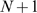 Gaussian model would be significantly better by performing 10-fold cross-validation. In each fold, 90% of the data was used to fit an 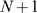 Gaussian model, which was scored by the mean (across data points) log likelihood of the remaining 10% of the data. We calculated the mean and standard deviation (across folds) of the 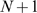 Gaussian model scores. If the mean was 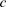 standard deviations greater than the score of the 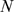 Gaussian model, we accepted the increase to 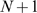 Gaussians, and performed the process again. We chose 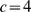, as we found this was sufficiently stringent to prevent splitting of what were clearly single subpopulations (e.g., at zero galactose concentration).

Mode estimation for the mode-estimation-plus-EM approach began by smoothing the data by taking a running average over a window of size 71 channels. Call this 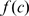. First and second derivatives, 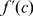 and 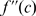, were estimated by computing centered finite differences, with the same width of 71 channels. A mode in the density was detected at channel 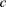 point if the first derivative crossed from positive to negative (i.e., 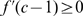 and 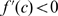) and if 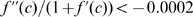. Ordinarily, one might threshold only the second derivative. However, small bumps in the data series are characterized by both smaller first and second derivatives in the vicinity of a mode, and combining them in this way lead to more robust and balanced detection of peaks of all sizes in preliminary tests. The choices of a 71-width averaging window and the −0.0002 threshold were based on pilot testing on a separate, but related, set of flow cytometry data.

For fitting the conditional mixture density models, we used only a single run of EM, as further runs did not improve accuracy. Updates are standard, as given in Bishop [35]. Low and high subpopulation means were initialized to have means of 200 and 700 respectively (independent of galactose level), standard deviations were initialized to 50, and mixture probabilities to 0.49.

### Availability

All code is written in MATLAB. Code and raw data are available upon request, as well as on TJP's website: http://www.perkinslab.ca
