# Correction: Estimating the Stochastic Bifurcation Structure of Cellular Networks

**DOI:** 10.1371/annotation/9a35fa58-f81c-41b4-9fa9-c1aeedbf0fff

**Published:** 2010-03-15

**Authors:** Carl Song, Hilary Phenix, Vida Abedi, Matthew Scott, Brian P. Ingalls, Mads Kærn, Theodore J. Perkins

In the Funding section there is an incorrect website address. The correct website can be viewed here: http://www.matrixpharma.ca

